# Acceptance of anomalous research findings: explaining treatment implausibility reduces belief in far-fetched results

**DOI:** 10.7717/peerj.12532

**Published:** 2021-11-23

**Authors:** W. Burt Thompson, Milen L. Radell

**Affiliations:** Department of Psychology, Niagara University, Lewiston, NY, United States of America

**Keywords:** Treatment plausibility, Research methodology, Statistics, College teaching

## Abstract

Research findings are best understood by considering contextual factors such as treatment plausibility: how likely it is that a studied treatment or manipulation is effective, based on theory and data. If a treatment is implausible, then more evidence should be required before believing it has an effect. The current study assessed the extent to which the interpretation of a research finding is affected by treatment plausibility. Participant age varied from 18 to 82 (*M* = 27.4, *SD* = 9.4), and about half of the participants (53%) were college students. A total of 600 participants read a brief news article about an experiment with a new type of psychotherapy for weight loss. The current study used a 2 (treatment plausibility) × 3 (results type) between-subjects factorial design. Treatment plausibility had two levels: (1) a plausible cognitive behavioral therapy and (2) an implausible ”psychic reinforcement therapy” that was described as employing psychic messages to promote weight loss. The three levels of the results type factor varied how the study results were presented in the article: (1) standard results with no mention of treatment plausibility, (2) standard results followed by interpretive statements focused on treatment plausibility, and (3) no results—the study was described as still in progress. Participants rated their belief in the effectiveness of the therapy on a scale of 0 to 100% in 10% increments. When treatment plausibility was not discussed in the article, average ratings for the implausible therapy were relatively high (*M* = 63.1%, *SD* = 25.0, 95% CI% [58.2–68.1]) and similar to those for the plausible therapy (*M* = 69.2%, *SD* = 21.5, 95% CI% [65.0–73.5]). Ratings for the implausible treatment were moderately lower when the article explained why the results supporting it were questionable (*M* = 48.5%, *SD* = 26.6, 95% CI% [43.2–53.8]). The findings of the current study suggest that students and other members of the public may draw incorrect inferences from research partly because they do not appreciate the importance of treatment plausibility. This could be remedied, though not completely, by explicitly discussing the plausibility of the treatment based on theory and prior data.

## Introduction

It is a truism that research results do not stand on their own. Science is a collaborative effort, and researchers must rely on each other’s work. This reliance is evident in every aspect of science, from the norm of publishing methods and findings to the long list of articles cited in the typical journal article, and was seemingly the basis for Isaac Newton’s famous remark, “if I have seen further it is by standing on the sholders [*sic*] of Giants” (Gleick, 2004, p. 98). Because of this collaboration, the findings of a scientific study are best understood by taking into account various aspects of the context in which they occur (*e.g.*, Lykken, 1968; Wasserstein & Lazar, 2016). A study may find a positive effect for a new treatment, but that result has little meaning by itself. Its interpretation is influenced by factors such as the adequacy of the study design. The current study focused on another crucial contextual factor—the credibility or plausibility of the treatment.

For example, severe test anxiety is fairly common, affecting perhaps one in four college students (Huntley et al., 2019). Researchers have conducted dozens of studies searching for effective treatments, and a variety of interventions have been tested. One study found, for example, that the treatment group “was significantly improved” compared to the control group after two months (Sezgin & Ozcan, 2009, p. 28). If you were a test anxious student, you might be willing to try the treatment, given this promising report. However, with further investigation you would discover that a key part of the treatment is to use your fingers to tap on your body in specific places—places based on traditional Chinese acupuncture. Does the treatment still sound promising, and would you still be eager to try it? Although responses will vary from person to person, this example illustrates the question that motivated the current study: how does a treatment’s plausibility affect the interpretation of research findings?

We use *treatment plausibility* to refer to a judgment about how likely it is that a study treatment or manipulation had (or will have) some proposed effect. That judgment combines two broad sources of information: data and theory. Preferably, there will be empirical evidence from previous evaluations of the treatment or other relevant evidence such as tests of similar treatments. However, there may be little relevant prior evidence to help interpret an unexpected result. As Matthews (2018, p. 5) pointed out, “Such ‘out of the blue’ findings commonly emerge from exploratory studies, and are perhaps most familiar in epidemiology, which is replete with claims of seemingly implausible causal connections between some environmental exposure and negative health effects.” Of course, “out of the blue” findings are also reported in other fields, including psychology. In this case, when direct prior evidence is not available, a plausibility judgment can be based on theory, informing the coherence of the proposed process by which the treatment produces an effect. David & Montgomery (2011) suggested using essentially the same two criteria—empirical support and theory—to evaluate psychological treatments. In this sense, treatment plausibility is analogous to the concept of biologic plausibility in epidemiology (Hill, 1965), which has been defined as “coherence with the body of biologic knowledge” (Celentano, Szklo & Gordis, 2018, p. 277).

Judgments of plausibility should play an important role in research evaluation. In particular, if a treatment is implausible, then more evidence should be required before believing it has an effect. Researchers occasionally state this principle explicitly. For example, (Quintana & Williams (2018, p. 2) wrote, “theoretically implausible claims should require more evidence than usual for their support.” Regarding Bem’s (2011) research supporting extrasensory perception (ESP), Rouder & Morey (2011) wrote, “ESP seems contradicted by well-substantiated theories in physics and biology. Consequently, it is reasonable to have low prior odds on ESP. In our view, while the evidence provided by Bem is certainly worthy of notice, it should not be sufficient to sway an appropriately skeptical reader.” And, in a discussion of pseudoscience, (Sagan, 1979, p. 62) wrote “I believe that the extraordinary should certainly be pursued. But extraordinary claims require extraordinary evidence.” If the proposed process by which a treatment produces an effect is implausible, then even a statistically significant finding could well be a false alarm (*i.e.*, a Type I error). The opposite might be true for a more plausible treatment. As Goodman (2008, p. 138) explained, considering theory and findings from prior work, “In some instances, a scientifically defensible conclusion might be that the null hypothesis is still probably true even after a significant result, and in other instances, a nonsignificant *P* value might still lead to a conclusion that a treatment works.” Thus, a single nonsignificant finding would not prevent a researcher from concluding that a treatment works if that finding is outweighed by other evidence that the treatment is effective.

### Treatment plausibility as prior odds

Viewing treatment plausibility as an estimate of prior odds or probability is one way to appreciate its importance for evaluating research evidence (Lilienfeld, 2011). Prior odds represent the level of belief or confidence one has, before study results are known, regarding whether a treatment works, or the extent to which it works. Prior odds in favor of a treatment effect are higher, for example, when previous research suggests the treatment might be effective, or when the way in which the treatment is believed to work is consistent with other accepted knowledge. Then, taking new data into account, the prior odds are updated via Bayes’ rule to yield posterior odds.

An underappreciated consequence of Bayes’ rule is that the initial level of belief can have a large influence on posterior odds. Two studies might produce the same result, but if one treatment is more plausible than the other, the posterior odds can be much higher for the plausible treatment. Thus, two treatments tested in essentially identical studies could yield the same positive results, yet it might be appropriate to accept that one treatment probably works while rejecting the other treatment as still unproven.

**Figure 1:**
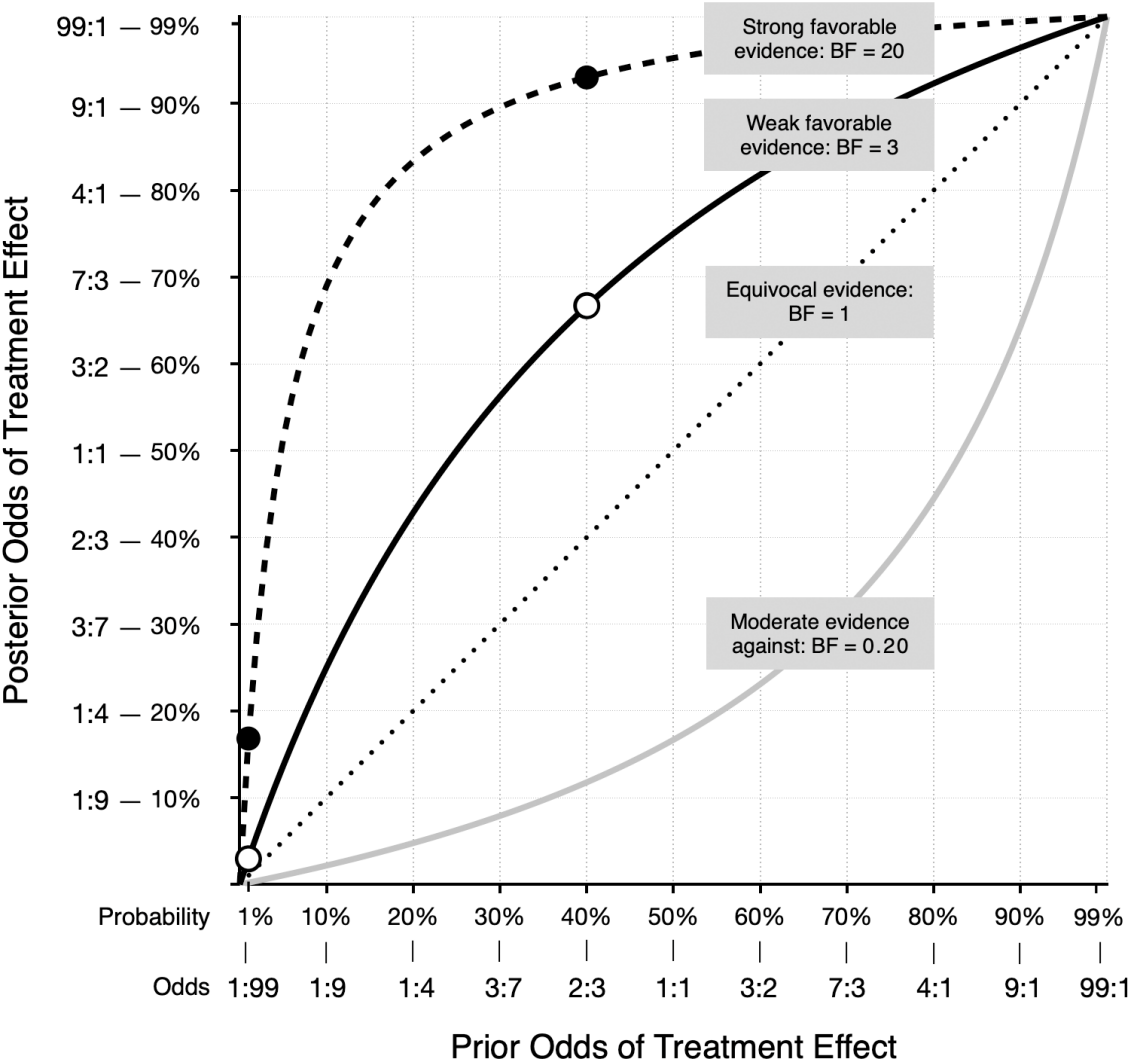
Posterior odds that a treatment is effective as a function of prior odds, for four types of evidence. The two white dots on the solid line, and the two black dots on the dashed line, represent the posterior odds for treatments with prior odds of 1:99 and 2:3.

This somewhat counterintuitive result is illustrated in Fig. 1. Derived using Bayes’ rule, Fig. 1 shows the relationship between the prior odds that a treatment is effective and the posterior odds, given four possible types of evidence from a new study. For example, suppose the prior odds that an implausible treatment works are just 1 to 99 (1:99 = 1/100 = 1%), while a much more plausible treatment has prior odds of 2:3 (= 2/5 = 40%). Each treatment is then evaluated in a well-designed study, and both studies yield the same weak evidence for a positive treatment effect. For this example, weak evidence is defined as a Bayes factor of 3 (Kass & Raftery, 1995). In other words, the probability of obtaining the study result is 3 times greater if the treatment works than if it does not work. An example of a classical *t*-test reflecting this amount of evidence is *t*(78) = 2.45, *p* = .017^1^. 1The *t*-test results that correspond to specific Bayes factors were obtained using the Summary Stats module in JASP (JASP Team, 2020), which is based on the BayesFactor R package (Morey & Rouder, 2015). The fictitious news articles state that the study used a sample size of 40 for each group, therefore we specified the same for our Bayes factor calculations. We also used a nondirectional test and the Cauchy default prior. An online Bayes factor calculator based on the BayesFactor package can be found at http://pcl.missouri.edu/bayesfactor.The solid line in Fig. 1 shows the prior-posterior function for a Bayes factor of 3. According to the odds form of Bayes’ rule, prior odds × Bayes factor = posterior odds. For the implausible treatment the odds increase from 1:99 to 3:99 (*i.e.*, from 1% to about 3%). Thus, despite the statistically significant result, it is still very unlikely the treatment has a positive effect. However, for the more plausible treatment, the same evidence increases the odds from 2:3 to 6:3 (from 40% to 67%). In Fig. 1, the two white dots on the solid line represent the posterior odds for the two treatments.

A qualitatively similar pattern occurs with much stronger evidence, such as a Bayes factor of 20, which reflects data that produce *t*(78) = 3.26, *p* = .002. The dashed line in Fig. 1 illustrates the prior-posterior function for this result. The black dots show the posterior odds for prior odds of 1:99 and 2:3. With strong evidence, the odds in favor of the plausible treatment experience a large increase: 2:3 × 20 = 40:3, which corresponds to a probability of about 93%. The same amount of evidence increases the odds for the implausible treatment from 1:99 to 20:99 or about 17%. In this case, the treatment remains implausible, despite results that some researchers might label “highly significant (*p* = .002).” Scientists should be open to new possibilities, of course, because seemingly implausible treatments may actually work, and promising plausible treatments may not. If more studies are conducted and positive evidence continues to accumulate for an initially implausible treatment—and that evidence is not offset by negative findings—then the treatment is no longer considered implausible. In Fig. 1, the gray line below the diagonal shows how moderate evidence *against* a treatment effect reduces the odds that the treatment works. However, if the prior odds are high, you may still have confidence in a treatment, even after new moderate negative evidence. Again, as with positive evidence, the function is not linear, and shows that the meaning of a study result can depend heavily on the treatment’s plausibility as expressed by prior odds.

The quantitative examples above show how a consideration of treatment plausibility aids the interpretation of research results. However, this process can be more informal and qualitative. As Motulsky (2018) writes, “Even if you don’t do formal Bayesian calculations, you should still consider prior knowledge and theory when interpreting data” (p. 178). Also, the basic application of Bayes’ rule—whether formal or informal—to help interpret research results should not be confused with the more involved process of Bayesian inferential statistics. Nor is it limited to the interpretation of Bayesian statistics. More simply, interpreting any research result can benefit from doing so in the context of prior knowledge. Even a casual reader with no knowledge of statistics can benefit from asking themselves, “Is this the only study that has come to this conclusion, or are there others?”

### Reasons for neglecting treatment plausibility

Why might students and other members of the general public fail to consider treatment plausibility when interpreting research findings? As discussed above, the relationship between prior odds and posterior odds is somewhat counterintuitive, and people may not adjust their beliefs as specified by Bayes’ rule (*e.g.*, Hilbert, 2012; Phillips & Edwards, 1966). Also, previous research suggests that people can give too much evidential weight to the results of a study they are currently reading (Thompson et al., 2020), which may reflect the operation of an availability bias (Tversky & Kahneman, 1973). If so, then they would be less influenced by contextual factors such as the results of previous research or the plausibility of the studied treatment.

Perhaps more fundamental is a lack of relevant domain-specific knowledge. Unlike specialists, the general public simply may not know whether it is more or less plausible that a particular manipulation or treatment will have an effect. Widespread misconceptions about many psychological topics are well-documented (Lilienfeld et al., 2011), including misconceptions about mental illness and its treatment (Basterfield et al., 2020). Also, research findings that confirm our beliefs may be more readily accepted, while unexpected findings are met with doubt (*e.g.*, Kaptchuk, 2003). This confirmation bias, together with a lack of knowledge, may result in people accepting an implausible result because it fits with a misconception they hold. Thus, it would not be surprising for some people to believe positive results for the finger tapping therapy, while knowledgeable specialists might be more skeptical (see Gilomen & Lee, 2015, for a review of so-called acupoint stimulation treatments). Indeed, one study found that experts rated an implausible finding as less important and less deserving of publication than a more plausible finding (Resch, Ernst & Garrow, 2000). A lack of knowledge may result in plausible and implausible treatments alike being assigned similar prior odds. As a result, whatever the study outcome, belief in both treatments may be adjusted up or down about the same amount.

A factor stemming from lack of knowledge is that writers may use specialized terms that impede clear communication, making it more difficult for members of the general public to interpret study results. Scientific jargon is necessarily a part of publications written by and for experts. But few people read journals—most get science information from general news sources (Funk, Gottfried & Mitchell, 2017). Scientific terms that appear in a news report may be misunderstood by many readers. For example, consider this passage from a recent news article (Reynolds, 2020): “After three months of working out, their overall scores on the depression scale fell by about 35 percent, a significant difference from the control group, whose depression scores had barely budged.” The term “significant” (presumably meaning “statistically significant”) will be taken by most people to mean that the difference is both real and important (Haller & Krauss, 2002; Tromovitch, 2015), and therefore the treatment must have worked. Another article (Bakalar, 2020) contains this passage: “There was no statistically significant difference in pain intensity between the treatment and the placebo groups at either time point.” In this example, the phrase “no statistically significant difference” may lead some readers to conclude that the treatment does not work. The use of widely misinterpreted terms may lead readers to draw the mistaken conclusion that a treatment does or does not work, regardless of the treatment’s plausibility or the width of the associated confidence interval.

The importance of treatment plausibility may also be overlooked if people tend to take descriptions of research findings at face value. In general, scientists enjoy a relatively high level of public trust (Funk et al., 2020). If a news article reports that a treatment was effective, many people accept that assessment. What may not be widely appreciated by members of the public is that scientific progress is uneven, advancing in fits and starts, and that any one research finding does not necessarily have a high probability of being correct.

### The current study

To summarize, research results cannot be interpreted correctly unless treatment plausibility is accounted for, but there are several possible reasons why this may not happen. There is some evidence that treatment plausibility affects the way experts evaluate evidence (Lykken, 1968; Resch, Ernst & Garrow, 2000), and that experts, as a group, can predict with fair accuracy whether social science research results can be replicated (Camerer et al., 2018). But there is a lack of research with people who are not experts. For this reason, the primary goal of the current study was to assess the extent to which students and other members of the general public are affected by treatment plausibility when interpreting study results. To accomplish this, we had participants read a brief science news article. The article described a study that tested the effectiveness of psychotherapy for helping people lose weight. The key manipulation was the plausibility of the therapy described in the article. Some participants read about a type of therapy that could plausibly help people lose weight: cognitive behavioral therapy. For the implausible therapy, we fabricated a treatment called psychic reinforcement therapy (hereinafter, psychic therapy).

We sought to answer three main questions. First, relative to a plausible treatment, how common is it to believe that an implausible treatment could work based on a single positive finding? If the belief is common, then instructors for statistics and research methods classes should consider helping their students learn the importance of considering treatment plausibility when trying to make sense of research findings.

Our second question concerned a simple but potentially effective way to highlight treatment plausibility: would ratings of treatment effectiveness be lower if a news article explained why the results for an implausible treatment should be viewed with skepticism? If so, it would suggest how researchers and writers might more clearly communicate the meaning of study findings. In this case, the news article would contain a bit of domain-specific knowledge that would help readers by providing important contextual information.

A third key question was whether statistical experience would moderate the relationship between treatment plausibility and ratings of therapy effectiveness. This is important because it relates to what people learn when they study statistics and research methodology, and to how writers and researchers explain research findings. It would be understandable if treatment plausibility does not have much of an effect on people with little knowledge of research and statistics. However, participants with more knowledge should be suspicious of positive results for an implausible treatment. If they are, it would be consistent with the idea that training helps students understand that plausibility is an important factor in their interpretation of research findings. If not, it would suggest an area where instruction might be improved.

## Materials & Methods

The study was approved by the Niagara University Institutional Review Board (#2020-054). Study materials and the preregistration document are available from the Open Science Framework website (https://osf.io/c4yhs/).

### Design and participants

The study used a 2 (treatment plausibility) ×3 (results type) between-subjects factorial design. Treatment plausibility had two levels: plausible and implausible. The three levels of the results type factor varied how the study results were presented in the article: (1) standard results with no mention of treatment plausibility, (2) standard results followed by several interpretive statements focused on treatment plausibility, and (3) no results—the study was described as still in progress. Participants were randomly assigned to conditions (*n* = 100 per condition). We conducted *a priori* power analyses for detecting differences between any two combinations of treatments. This sample size yielded statistical power of 94% using traditional power analysis (Cohen, 1988), assuming a two-sided alpha level of .05 and a moderate effect (*d* = 0.50). A Bayesian design analysis (Stefan et al., 2019) indicated a 79% probability of obtaining a Bayes factor larger than 10.

Participants were recruited through the online research platform Prolific (https://www.prolific.co) and from classes at Niagara University, a private liberal arts university located in the northeastern United States. Participants represented 47 nationalities and were fluent in English. Participants recruited via Prolific (*n* = 579) received a payment of $1.25 for completing the study, and the students at our university (*n* = 21) received research participation credit in their classes. Participant age ranged from 18 to 82 (*M* = 27.4, *SD* = 9.4), and 54% reported their gender as male. Just over half (53%) of all participants were students. Of the 189 participants between the ages of 18 and 21, 90% were students.

### Materials and procedure

The study was titled “News Article Comprehension and Interpretation,” and participants were told “the general purpose of the study is to learn more about how people interpret news articles.” After agreeing to a written consent statement, participants were randomly assigned to read one of six articles. The articles were modeled on the first few paragraphs of an actual news article (Sample, 2020). We chose cognitive behavioral therapy as our plausible treatment because research shows that it can be effective at helping people lose weight. For a fictitious implausible treatment, we created a brief description of therapy based on the alleged psychic phenomenon of telepathy.

**Figure 2:**
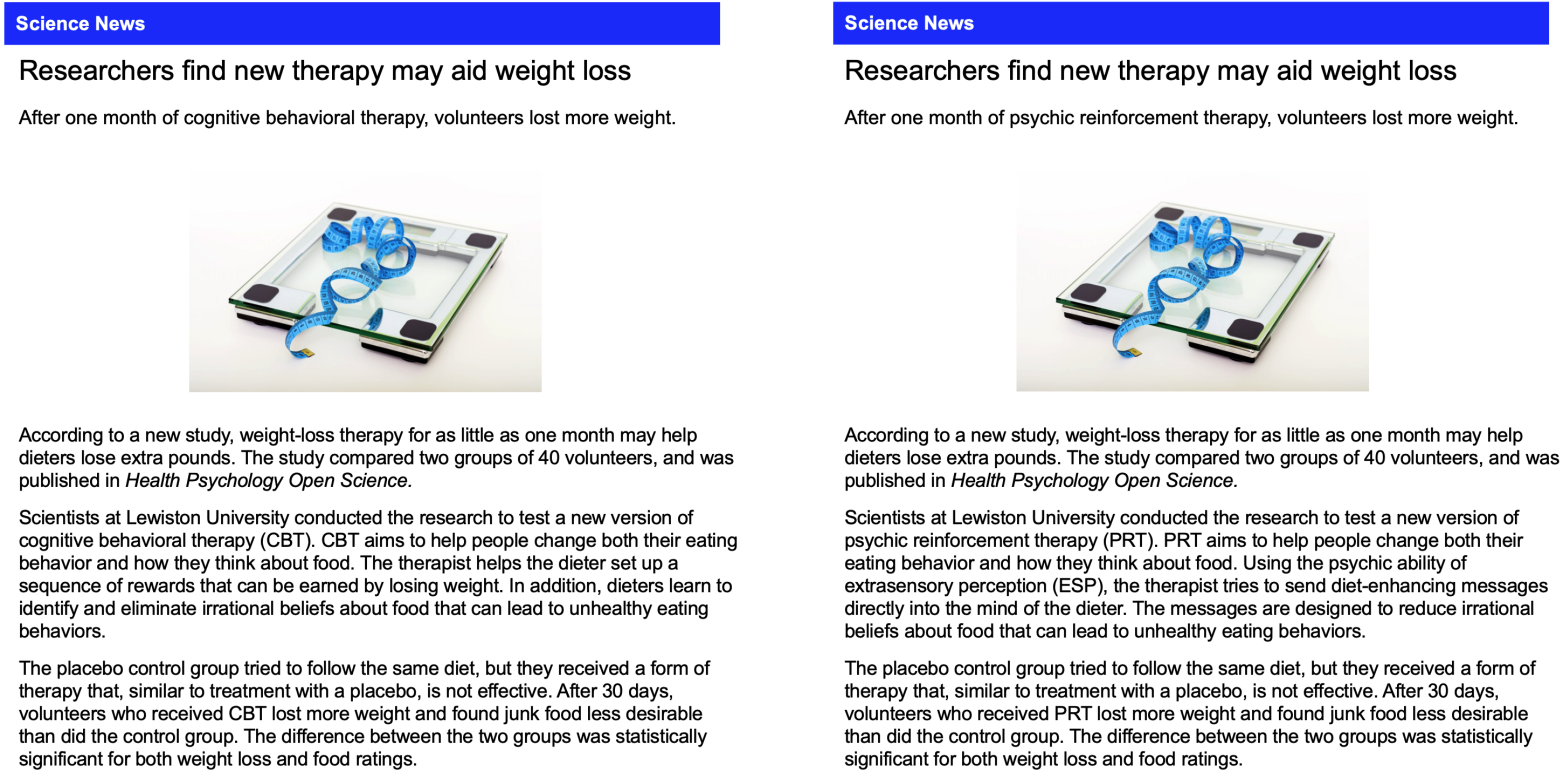
Articles with standard results for the plausible and implausible treatment.

The six articles can be found in the materials on the Open Science Framework website (https://osf.io/c4yhs/). Figure 2 shows the articles that contain the *standard results* presentation for the plausible treatment (cognitive behavioral therapy) and the implausible treatment (psychic therapy). We labeled these results “standard” because they are typical of brief news articles that describe scientific studies. Such articles often describe findings with appropriate hedge words (“new therapy may aid weight loss”) and use jargon like “placebo” and “statistically significant.” Because the articles are brief, they provide minimal contextual information for evaluating the findings. For the *no-results* condition, the study was presented as still in progress, and the article described the measures and analyses that were planned:

After 30 days researchers will measure which group lost more weight and also assess their desire to eat junk food. The researchers will analyze the study data to determine if the difference between the two groups is statistically significant for both weight loss and food ratings.

For the *interpretive results* condition, additional information was included at the end of the article. This information was presented as a quote from the researchers, and explained how they used information about treatment plausibility to interpret the study results. The statements were intended to represent the reasoning that would be typical of researchers who obtain positive results for a plausible treatment and an implausible treatment. For the plausible condition (cognitive behavioral therapy), this was the additional information:

The outcome of the study was not a surprise to the researchers. They noted that “cognitive behavioral therapy is based on sound, widely accepted scientific principles. That’s why we thought the therapy would help people stick to a diet. Statistically significant differences can occur just by chance, even when a therapy is not effective, but that probably didn’t happen in our study. There is good reason to believe the therapy should work, so we think the results are probably not a fluke. But, additional research will help us answer that question with more certainty.”

For the implausible condition (psychic therapy), this was the additional information:

The outcome of the study was a surprise to the researchers. They noted that “psychic reinforcement therapy is not based on sound, widely accepted scientific principles. That’s why we thought the therapy would not help people stick to a diet. Statistically significant differences can occur just by chance, even when a therapy is not effective, and that is probably what happened in our study. There is no good reason to believe the therapy should work, so we think the results are probably just a fluke. But, additional research will help us answer that question with more certainty.”

After giving consent, participants saw these instructions: “Please read the following news article carefully, then answer the questions that follow. The article was created specifically for this study. It does not describe actual events. However, please answer the questions as if the article describes actual events.” After reading the article, participants responded to 27 items (see Supplemental Information). The article and items 1–10 were presented together on one page so participants could refer back to the article if desired. Items 1–6 tested article comprehension and were used to verify that participants understood basic facts stated in the articles.

The main dependent measure was assessed with item 7. Using an 11-point scale (0% to 100%), participants indicated how likely it was that the therapy helped people lose weight. Item 8 asked for a brief explanation for the answer to item 7. Item 9 asked participants if they would be willing to try the therapy, and item 10 asked if they would recommend it to a friend. Both items were answered using a response scale that was scored from 0 to 4, and the sum of the scores constituted a *therapy application rating* that could vary from 0 to 8. The answers to items 13–17 (from Thompson et al., 2020) were combined into a total *statistics experience* score. Each answer was assigned a score from 0 to 4 (items 13–14) or 0 to 3 (items 15–17), with higher scores indicating more statistical experience, and total scores could range from 0 to 17.

Items 11–12 were included as exploratory measures. They asked participants to rate how often they read scientific or medical research articles and how often they have dieted to lose weight. We assessed two other variables for exploratory purposes. Items 20, 21, 24, and 27 (from Schofield et al., 2018) assessed belief in psychic phenomena. Answers were converted to a 0–5 scale (item 21 was reversed scored) and summed. These *psychic belief scores* could range from 0 to 20, with higher scores indicating stronger belief in psychic phenomena. Similarly, the answers to items 22, 23, 25, and 26 were totaled (reversing 25 and 26) to create a *therapy belief score* that could range from 0 to 20.

## Results

Data are available on the Open Science Framework website (https://osf.io/c4yhs/). As specified in the preregistration, we restricted our analysis to the first 100 participants assigned to each condition who correctly answered at least 5 of the 6 news article comprehension items. The analyses of therapy effectiveness ratings, statistics experience, and therapy application rating were confirmatory, meaning the analyses were specified before data collection began (Wagenmakers et al., 2012). Exploratory analyses were conducted with psychic belief scores and therapy belief scores.

### Therapy effectiveness ratings

**Figure 3:**
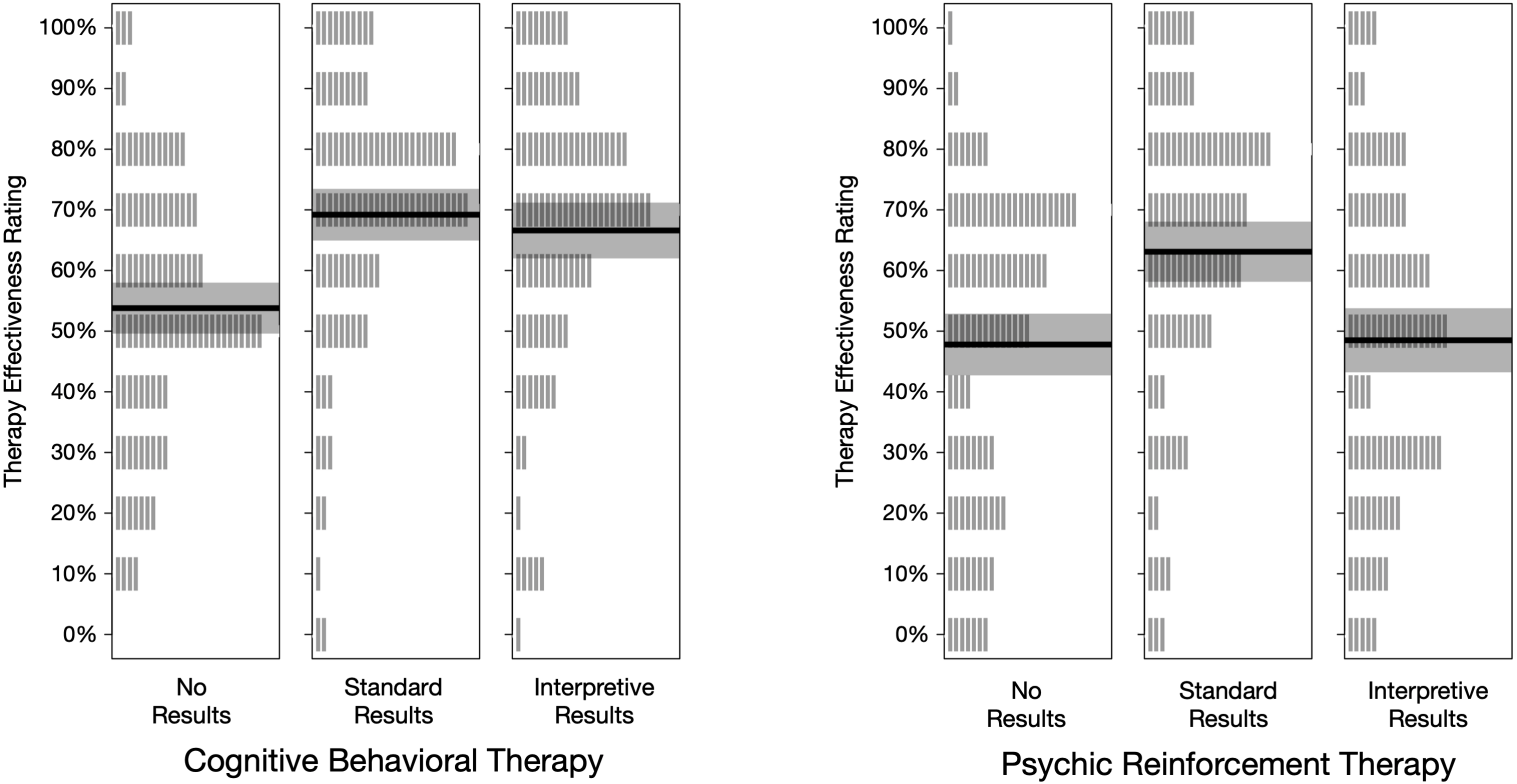
Treatment effectiveness ratings, by condition. Each vertical gray line represents one rating (*n* = 100 per condition). The height of the shaded rectangle represents the 95% credible interval, and the solid line is located at the group mean.

Our primary dependent measure was the answer each participant gave to item 7: “Based on the information in the article, how likely do you think it is that the therapy helped people lose weight?” Participants chose from 11 response options: 0% to 100% in 10% increments. These *therapy effectiveness ratings* are summarized in Table 1 and graphed in Fig. 3. Figure 3 shows that the mean rating was near 50% for both types of therapy when the articles contained no results. Ratings were higher under standard results, and lower for psychic therapy under interpretive results.

**Table 1:** Summary statistics for therapy effectiveness ratings (0% to 100%), by experimental condition. Therapy effectiveness ratings were made using an 11-point scale that ranged from 0% to 100% in 10% steps.

		*M*	*SD*	N	95% Credible interval
Cognitive behavioral therapy					
	No results	53.8	21.2	100	49.6 to 58.0
	Standard results	69.2	21.5	100	65.0 to 73.5
	Interpretive results	66.6	23.2	100	62.0 to 71.2
Psychic reinforcement therapy					
	No results	47.8	25.6	100	42.8 to 52.9
	Standard results	63.1	25.0	100	58.2 to 68.1
	Interpretive results	48.5	26.6	100	43.2 to 53.8

**Figure 4:**
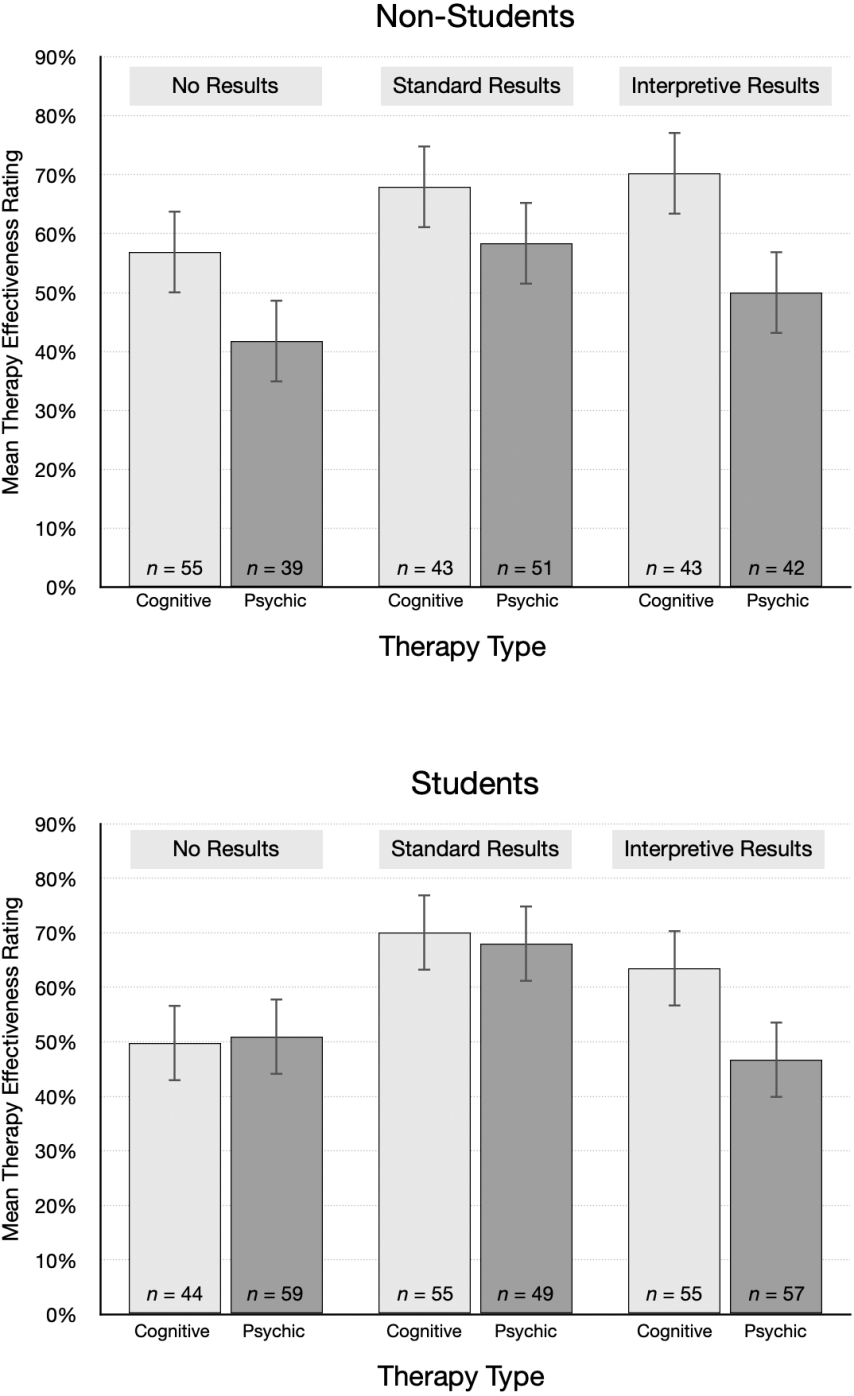
Mean treatment effectiveness ratings (95% CI), by condition and student status.

The pattern of mean ratings was different for non-students and students, as shown in Fig. 4. The average rating from non-students for psychic therapy was lower than cognitive behavioral therapy for each type of results. In contrast, students’ effectiveness ratings were essentially the same for psychic therapy and cognitive behavioral therapy in the no results and standard results conditions. Ratings were also surprisingly high. For example, after reading the standard results article, students gave an average rating of 70.0% to cognitive behavioral therapy and 68.0% to psychic therapy. On average, non-students were 10 years older and had attained a higher educational level than students, which we speculate may account for the different patterns. However, non-students were not highly skeptical of the implausible treatment: even after reading interpretive results, students and non-students alike gave fairly high average ratings to psychic therapy (*M*s = 46.7% and 50.0%, respectively).

To analyze the ratings we used JASP (JASP Team, 2020) to conduct a 2 × 3 Bayesian ANOVA (Rouder et al., 2012). One advantage of the Bayesian approach is that it specifies how the relative probabilities of contrasting models change, given the observed data (*e.g.*, Kass & Raftery, 1995; Wagenmakers et al., 2018). Also, unlike classical inferential statistics, Bayesian inference can quantify, in the form of a Bayes factor, the evidence for or against hypotheses. This allows researchers to specify, for example, the strength of the evidence in favor of the null hypothesis relative to the alternative hypothesis. In the current study, we used the guidelines in Kass & Raftery (1995) for interpreting the strength of Bayes factors. For example, we refer to Bayes factors near 1 as equivocal, indicating they provide little evidence for or against the alternative hypothesis relative to the null hypothesis. Bayes factors between 20 and 150 are labelled as strong evidence, while those greater than 150 are considered very strong evidence.

To conduct the ANOVA we used the default prior settings in JASP. Table 2 shows the summary of model comparisons, in descending order of plausibility. The *P*(M) column shows that each of the five models was assigned a prior probability of .200. *P*(M—data) is the posterior model probability, that is, the probability after observing the data. The model with the highest posterior probability consists of both independent variables (treatment plausibility and results type) and their interaction. The Bayes factor for this model is BF_M_ = 6.78, meaning that the posterior model odds increased by a factor of 6.78 over the prior model odds.

**Table 2:** Models for therapy effectiveness ratings: prior probabilities, posterior probabilities, and Bayes factors.

Model	*P* (M)	*P* (M — data)	BF_M_	BF_10_
therapy type + results type + therapy type × results type	.20	.63	6.78	1.00
Therapy type + Results type	.20	.37	2.40	0.59
Results type	.20	1.40 × 10^−5^	5.58 × 10^−5^	2.22 × 10^−5^
Therapy type	.20	1. 08 × 10^−7^	4. 31 × 10^−7^	1. 71 × 10^−7^
Null model	.20	8.50 × 10^−12^	3.40 × 10^−11^	1.35 × 10^−11^

Table 3 summarizes the model-averaged results. The *P*(incl) column lists the prior probability for all models that include the effect. The *P*(incl—data) column lists the posterior probability for all models that include the effect. The inclusion Bayes factor (BF_incl_) quantifies the amount of evidence for including the effect in the model, and represents the degree to which the posterior model odds change from the prior odds in light of the data.

**Table 3:** Prior probabilities, posterior probabilities, and inclusion Bayes factors for each effect on therapy effectiveness ratings.

Effects	*P* (incl)	*P* (incl — data)	BF_incl_	
Therapy type	.60	>.99	4.78 × 10^4^	
Results type	.60	>.99	6.19 × 10^6^	
Therapy type × Results type	.20	.63	6.78	

The inclusion Bayes factors are very large for therapy type (47,782) and results type (6,191,000). They indicate the observed data are much more likely to occur under a model that includes those factors, relative to models that omit them. In contrast, the evidence is much weaker for including the Therapy Type × Results Type interaction (BF_incl_ = 6.78). In sum, this analysis reveals very strong evidence in favor of a model that includes both therapy type and results type, but support for inclusion of the interaction effect is relatively modest.

To supplement this analysis, we conducted an exploratory Bayesian ANCOVA that included therapy type and results type, with covariates of psychic belief score, therapy belief score, and statistics experience, to explore their relationship to the ratings. The prior probability, *P*(M), assigned for all models was .025. The model with the highest posterior probability, *P*(M—data) = .75, consisted of both independent variables, their interaction, psychic score, and therapy score, but not statistics experience. The Bayes factor for this model was BF_M_ = 115.77, meaning that the posterior model odds increased by a factor of 115.77 over the prior model odds. Table S1 summarizes the model-averaged results. Overall, based on the inclusion Bayes factors, there was very strong evidence in favor of a model that includes therapy type, results type, and psychic belief score but support for inclusion of therapy belief score and the interaction between the independent variables was relatively modest. There was no evidence for inclusion of statistics experience. To compute effect sizes, we also conducted a frequentist ANOVA with and without these covariates (see Tables S2 and S3). The pattern of results was the same as in the Bayesian analysis, with the largest effect size for psychic score (*η*_p_^2^=0.10). The effect sizes for therapy type, results type, and their interaction were similar, regardless of whether the covariates were included or not (*η*_p_^2^=0.05 vs. 0.04, respectively, for therapy type; 0.06 vs. 0.07 for results type, and 0.02 vs. 0.01 for the interaction). The effect size for therapy belief score was *η*_p_^2^=0.02, and that for statistics experience was near zero. The covariates are examined in more detail below.

**Figure 5:**
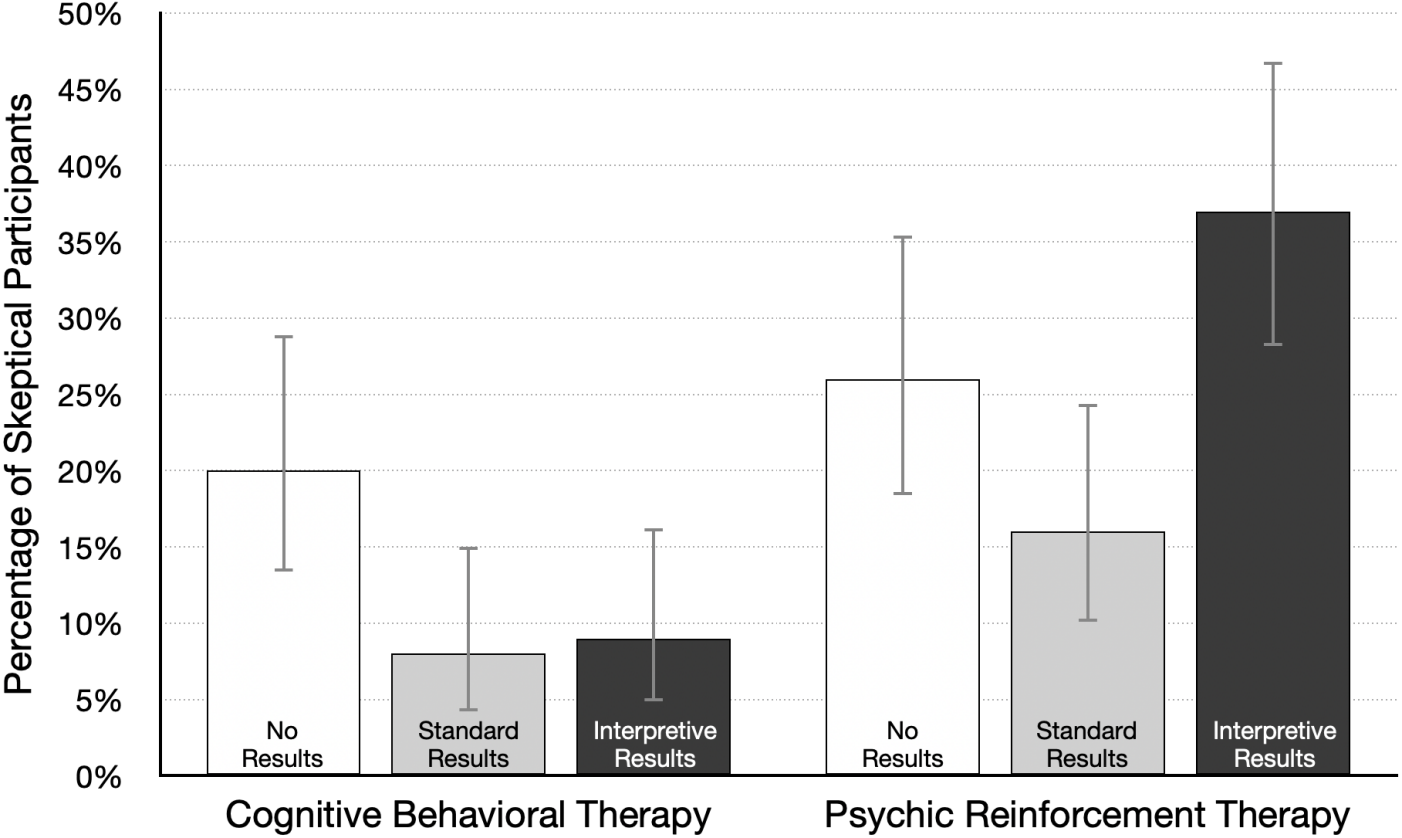
Percentage of participants skeptical of therapy effectiveness (95% CI), by condition. A skeptical participant was defined as one who gave a treatment effectiveness rating of 30% or lower; *n* = 100 per condition.

First, we conducted Bayesian *t*-tests in JASP to investigate the effect size for key paired comparisons linked to our main research questions. As an exploratory adjunct to analysis of mean ratings, we also looked at the number of participants in each condition who were skeptical that the treatment was effective (or could be effective, in the no-results condition). A skeptical participant was defined as one who gave an effectiveness rating of 30% or lower. Those data are summarized in Fig. 5.

Our main question was whether participants would take treatment plausibility into account when interpreting study results. We first determined if one type of therapy was considered more plausible than the other. Because positive results might affect perceptions of plausibility, we compared ratings from participants in the no-results condition. In that condition participants were asked “Based on the information in the article, how likely do you think it is that the therapy will help people lose weight?” As shown in Table 1, the mean ratings for psychic therapy and cognitive behavioral therapy were 47.8% and 53.8%, respectively. A Bayesian *t*-test revealed that the data are equivocal, with a Bayes factor near 1: *d* = 0.24, 95% CI [−0.03, 0.51], BF_10_ = 0.70. This result does not provide convincing evidence for or against the null hypothesis, as both forms of therapy received average ratings near 50%. This is also reflected in the fact that just 20% of participants gave the plausible treatment a low rating (30% or lower), and 26% gave the implausible treatment a low rating (see Fig. 5). These data suggest that neither form of therapy is viewed as highly implausible, although cognitive behavioral therapy may be judged as slightly more plausible than psychic therapy.

We next examined how participants interpreted positive results reported in the standard format. In particular, we looked at whether effectiveness ratings of both treatments were higher relative to the no-results ratings. The mean ratings for psychic therapy and cognitive behavioral therapy were 63.1% and 69.2%, respectively (Table 1), and a Bayesian *t*-test revealed an equivocal Bayes factor close to 1: *d* = 0.17, 95% CI [−0.02–0.37], BF_10_ = 0.70. These results indicate that if there is an effect, it is likely to be small and in favor of cognitive behavioral therapy. As Fig. 2 shows, the highest average effectiveness rating for each type of therapy occurred in the standard results condition. In fact, 70% and 80% were the two most common ratings given to both therapies. Table 1 shows the mean rating was 15% higher under no-results, relative to standard results, for both types of therapy. This difference is also reflected in Fig. 5: fewer participants were skeptical of each therapy. These ratings may be partly based on the trust that people generally have in scientists. In the psychic therapy condition, the large number of high effectiveness ratings may also reflect the fact that belief in paranormal phenomena is fairly common. A 2018 survey of US adults found that 41% believe in psychics (Gecewicz, 2018). Likewise, the high ratings for both treatments are consistent with research showing widespread belief in the effectiveness of psychotherapy (Angermeyer et al., 2017). In sum, presenting results in a standard format led to relatively high average effectiveness ratings for the plausible cognitive behavioral therapy. However, it is somewhat worrying that ratings were nearly as high for psychic therapy, which was said to rely on ESP to “send diet-enhancing messages directly into the mind of the dieter.”

Our other central question was about the effect of an interpretive explanation for the positive results reported for psychic therapy. In that version of the news article, the researchers state (a) “Statistically significant differences can occur just by chance, even when a therapy is not effective,” (b) psychic therapy “is not based on sound, widely accepted scientific principles,” and (c) “we think the results are probably just a fluke.” How much did this explanation affect effectiveness ratings? To answer this question we compared the standard and interpretive results conditions. As Table 1 shows, the average rating was lower for standard results (63.5%) relative to interpretive results (49.6%), *d* = 0.42, 95% CI [0.22–0.62], BF_10_ = 715. Figure 5 shows that interpretive results more than doubled the number of skeptical participants (from 16% to 37%). These data show that the brief interpretive explanation reduced confidence in psychic therapy, and the size of the effect is likely in the small to medium range. But many participants still gave psychic therapy a rating of 50% or higher, even after reading that the researchers themselves doubted the results.

In contrast, interpretive results had little or no effect on ratings for the plausible treatment. The explanation for the cognitive behavioral therapy results included the statement “There is good reason to believe the therapy should work, so we think the results are probably not a fluke.” This is information that might increase confidence in the treatment. However, the mean rating without the interpretive explanation (in the standard results condition) was already very high at 69%. Moreover, as Fig. 5 shows, just 8% of participants were skeptical of cognitive behavioral therapy in the standard condition. Together, these data suggest that effectiveness ratings may have approached a ceiling in the standard results condition, leaving little room for improvement following interpretive results. A reviewer pointed out another possibility—researchers who are skeptical of their own findings may dampen plausibility, but talking up findings may not increase plausibility, perhaps because readers expect scientists to argue in favor of their findings.

### Statistics experience

One of our main goals was to learn if the relationship between treatment plausibility and ratings of therapy effectiveness would depend on the amount of statistical experience reported by participants. Statistics experience scores were positively skewed and ranged from 0 to 16 (mode = 4, *M* = 6.0, *SD* = 3.7). Internal reliability for our sample was good (McDonald’s *ω* = .85). On item 14, 39% responded “I know little or nothing about statistics” and an additional 29% said they have “an elementary knowledge of statistics.” Only 12% claimed to be either very knowledgeable or a statistical expert.

Participants with more statistics experience might be more likely to consider treatment plausibility when interpreting study results. However, based on the supplemental Bayesian ANCOVA, there was no evidence that statistics experience is related to therapy effectiveness ratings (Table S1). We also examined the correlation between these variables in each experimental condition. In the cognitive behavioral therapy condition, the correlations for no results, standard results, and interpretive results were .03, .14, and −.15, respectively. Similar weak correlations occurred for the three results types in the psychic therapy condition, .02, .03, and −.13, respectively. Even the strongest of these correlations (−.15, BF_10_ = 0.37) provides little evidence of a linear relationship.

**Figure 6:**
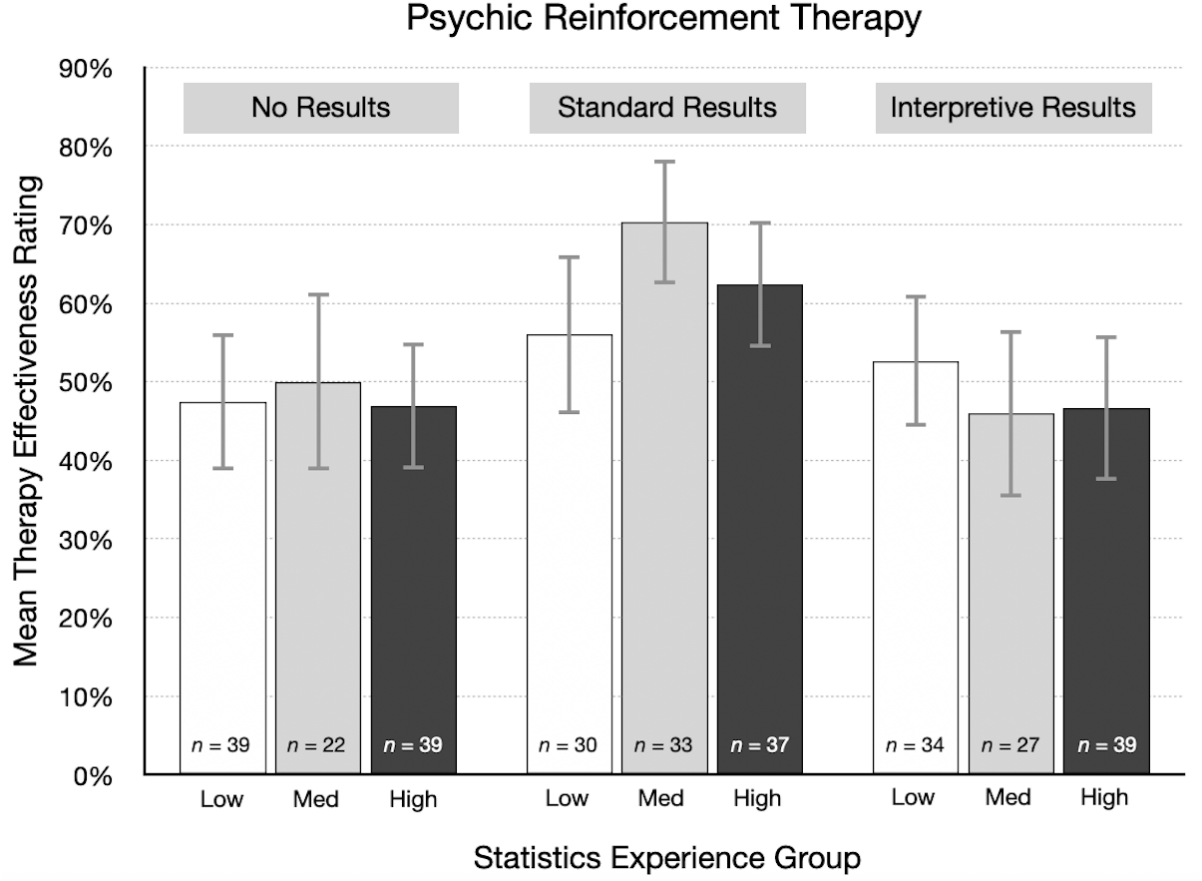
For each result type in the psychic therapy condition, mean therapy effectiveness ratings (95% CI) for participants with low, medium, and high statistics experience scores.

As one way of visualizing these results, participants were divided into three groups based on their statistics experience score: low (0–4, *n* = 227), medium (5–7, *n* = 163), and high (8–16, *n* = 210). We then summarized therapy effectiveness ratings in each experimental condition by statistics experience group. Figure 6 shows the results for the psychic therapy condition. As indicated by the correlations in the previous paragraph, there was little or no relationship between effectiveness ratings and statistics experience scores. For standard results, the average effectiveness rating was actually higher for participants in the high experience group (*M* = 62.4%) than for those in the low group (*M* = 56.0%). These results are not consistent with the idea that participants with more training and experience in statistics are more likely to doubt positive findings for an implausible treatment. A qualitatively similar pattern occurred in the cognitive behavioral therapy condition. However, it is important to note that statistics experience was not a manipulated variable, so a causal interpretation of this result is not justified in this particular instance.

### Therapy application ratings

After participants rated therapy effectiveness, they answered two questions that we combined into a *therapy application rating*. Ratings could vary from 0 to 8 (see Supplemental Information, questionnaire items 9–10) and reflected participants’ willingness to use and recommend the therapy. The ratings are summarized in Table 4. We assessed application because people do not just read science news articles for new information: they may also apply that information, putting it to use in their own lives. Application ratings are conceptually related to effectiveness ratings because the decision to use or recommend a therapy presumably depends in part on a judgement of effectiveness. If you believe psychic therapy works, you are presumably more likely to try it and more likely to recommend it to a friend. However, even if you believe psychic therapy works, you may prefer to use and recommend a different therapy that you believe is more effective. Thus, we expected application ratings to be positively related to effectiveness ratings, yet distinct enough to warrant the inclusion of both.

**Table 4:** Summary statistics for therapy application ratings, by condition. Therapy application ratings could range from 0 to 8, and are based on the responses to questionnaire items 9–10 (see Supplemental Information).

		*M*	*SD*	N	95% Credible interval
Cognitive behavioral therapy					
	No results	6.9	2.2	100	6.4 to 7.3
	Standard results	7.2	2.2	100	6.8 to 7.7
	Interpretive results	7.0	2.2	100	6.6 to 7.4
Psychic reinforcement therapy					
	No results	5.9	2.7	100	5.4 to 6.4
	Standard results	6.4	2.5	100	5.9 to 6.9
	Interpretive results	5.7	2.4	100	5.2 to 6.2

Like effectiveness ratings, therapy application ratings were first analyzed with a Bayesian ANOVA. The analysis showed that the model with therapy type was most plausible (BF_M_ = 17.5). Analysis of effects showed very strong support for including therapy type in the model (BF_incl_ = 56,840). As Table 4 shows, average application ratings were consistently higher for cognitive behavioral therapy. A Bayesian *t*-test revealed this effect size is likely to be moderate, *d* = 0.43, 95% CI [0.27–0.59]. In contrast, the data did not support including results type (BF_incl_ = 0.15) or the Therapy Type × Results Type interaction (BF_incl_ = 0.04) in the model. These analyses show, across all results types, that application ratings were somewhat lower for psychic therapy. This pattern differs from that observed for the effectiveness ratings, for which there was also evidence for including the interaction of therapy type and results type.

Importantly, average therapy application ratings, like effectiveness ratings, were relatively high for the plausible treatment and the implausible treatment. For example, in the condition that combined psychic therapy and standard results, the average rating was 6.4, which is 80% of the highest possible score of 8. In the condition that combined cognitive behavioral therapy and standard results, the average rating was 7.2 or 90% of the maximum.

### Exploratory analyses: therapy belief and psychic belief

As described in the Materials and Methods section, we measured several variables for exploratory reasons. Here we focus on therapy belief scores and psychic belief scores because they relate directly to our primary research questions. Based on the supplemental Bayesian ANCOVA, there was some evidence for a relationship between therapy belief scores and therapy effectiveness ratings, and stronger evidence for psychic belief scores (see Table S1). The effect sizes from the frequentist ANCOVA were *η*_p_^2^=0.02 and 0.10, for therapy belief and psychic belief scores, respectively (see Table S2). We also conducted Bayesian ANOVAs to determine if results type, therapy type or their interaction could predict therapy or psychic belief scores, which would suggest that there was an imbalance in these covariates between groups in the study. However, there was no evidence for this, making it less likely that any chance differences between treatment groups in terms of these variables could affect estimates of between treatment group differences (see Tables S4 and  S5).

Therapy belief scores were negatively skewed, and the items showed moderate internal consistency (McDonald’s *ω* = .64). The median score was 16 (*M* = 15.8, *SD* = 3.0), equivalent to an average scale rating of “agree.” As in prior research (Angermeyer et al., 2017), these scores show most participants think psychotherapy is effective. For example, 96% agreed to some extent with item 22 that therapy “can often help people deal with behavioral and psychological problems.” Participants who are more confident in the effectiveness of therapy may be more likely to give either form of therapy a higher effectiveness rating, and this is what we observed. There was an overall weak positive correlation between therapy belief scores and effectiveness ratings, *r* = .16, 95% CI [.09–.24]. The association was weaker for participants who read the no results article (*r* = .04) and the interpretive results article (*r* = .08). However, for the standard results article, the correlation was stronger, *r* = .37, 95% CI [.24–.48].

Psychic belief scores were roughly symmetrically distributed. The scale items exhibited good internal reliability (McDonald’s *ω* = .81). The average score (*Mdn* = 10, *M* = 9.4, *SD* = 4.4) was in the middle of the rating scale, between “slightly disagree” and “slightly agree.” This suggests that belief in psychic phenomena is fairly common, which is consistent with recent surveys (*e.g.*, Gecewicz, 2018). For example, 26% of participants agreed to some extent that telepathy exists and 52% agreed to some extent with the statement that many people have ESP. Participants with higher psychic belief scores might be expected to more readily accept the possibility that psychic therapy could work, and this would lead to higher effectiveness ratings. Overall, there was a moderate positive correlation between psychic belief scores and therapy effectiveness ratings, *r* = .29, 95% CI [.21–.36]. The correlation was weaker in the cognitive behavioral therapy condition, *r* = .16, 95% CI [.05–.27], but stronger in the psychic therapy condition, *r* = .43, 95% CI [.33–.52].

**Figure 7:**
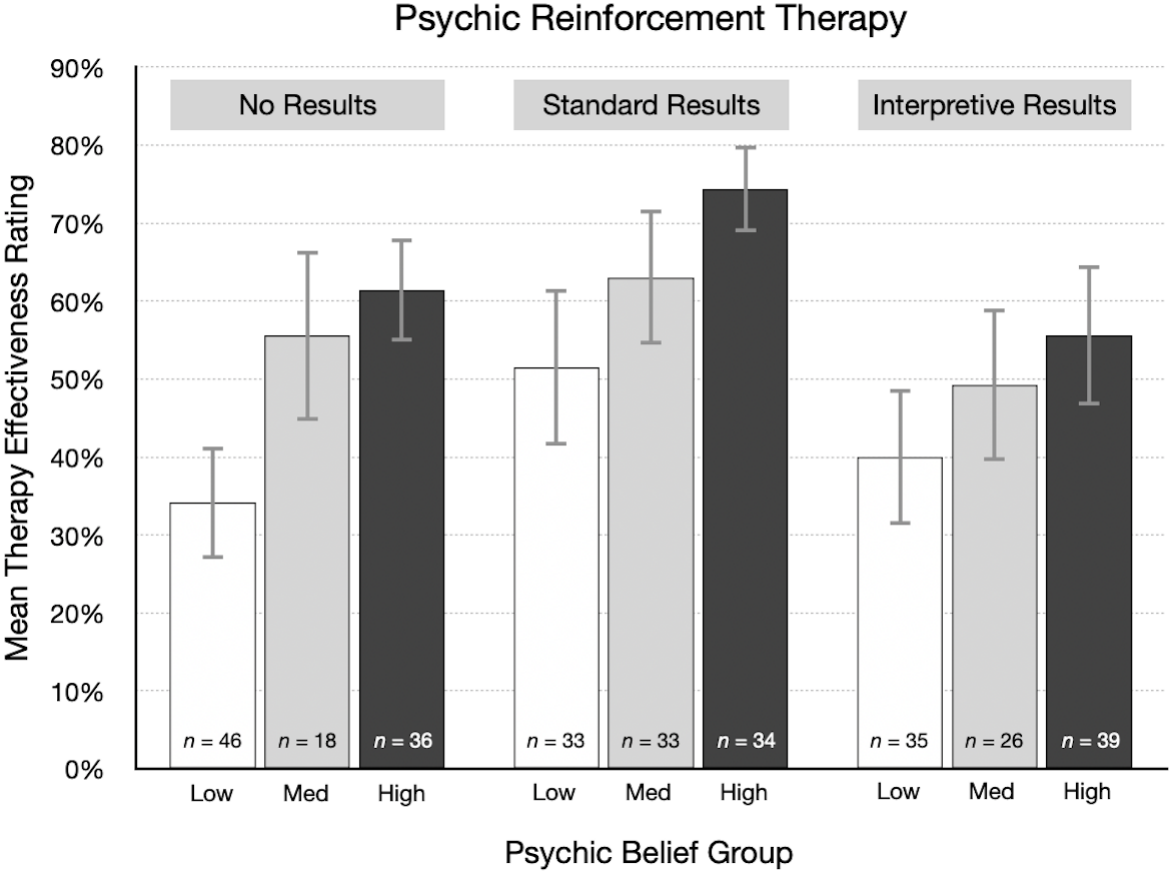
For each result type in the psychic therapy condition, mean therapy effectiveness ratings (95% CI) for participants with low, medium, and high psychic belief scores.

As a descriptive analysis, participants were divided into three groups based on their psychic belief scores: low (0–8, *n* = 228), medium (9–11, *n* = 165), and high (12–20, *n* = 207). Therapy effectiveness ratings were then summarized for each group. Figure 7 shows the mean rating for the three types of results in the psychic therapy condition. The direct relationship between psychic belief scores and effectiveness ratings is clear: participants with the highest psychic belief scores gave the highest average ratings. Also, many participants indicated that psychic therapy has a reasonable chance of working. This would be expected from participants who believe in ESP and psychics. Conversely, from participants who are skeptical of ESP, we would expect many very low effectiveness ratings, perhaps in the 0% to 20% range, but that did not happen. As Fig. 7 shows, the mean ratings from skeptical participants were surprisingly high. Particularly after reading standard results, psychic therapy was rated as having a good chance of working, even by many psychic nonbelievers. The large difference between the articles with no results (34%) and standard results (52%) suggests that the “statistically significant” finding is given too much attention, while prior beliefs receive too little.

## Discussion

Judgments of treatment plausibility should play an important role in the evaluation of research findings. Further, Bayes’ rule indicates that more evidence is needed to demonstrate that an implausible treatment is effective, compared to a plausible treatment. Prior research suggests that experts do consider plausibility (*i.e.*, consistency with theory and prior research) when judging whether a treatment has the effect reported in a scientific study (Alister et al., 2021; Lykken, 1968; Resch, Ernst & Garrow, 2000). However, research with nonexperts is lacking, thus the present study had students and other members of the general public rate treatment effectiveness for plausible and implausible treatments. The main findings were: (a) the plausibility of the treatment had little effect on treatment effectiveness ratings; (b) ratings decreased moderately when the reasons for implausibility were explained in the article; and (c) there was little or no association between self-reported statistical experience and ratings of treatment effectiveness. These results still held after accounting for beliefs in therapy effectiveness and in psychic phenomena.

One aspect of our findings that is important to highlight is that a relatively small fraction of participants expressed skepticism for the implausible treatment, psychic therapy. Given that about 50% of our participants did not believe in ESP and psychics, about 50% should have given psychic therapy very low ratings. However, even after reading that the researchers who conducted the study said the results were “probably just a fluke,” two-thirds of the participants gave the therapy an effectiveness rating of 40% or higher. Moreover, just 5% of participants gave psychic therapy an effectiveness rating of 0%, which we think is the most accurate assessment—the next response option, 10%, seems far too high. In comparison, only 1% of participants in the cognitive behavioral therapy condition gave it a rating of 0%. These data suggest that a few more participants did correctly judge psychic therapy to be highly implausible compared to cognitive behavioral therapy. However, we think the more striking result is the similarity in the ratings given to the two types of therapy in the no-results and standard results conditions. Those data are a reminder of an important lesson for students: contextual information in the form of domain-specific knowledge is important for correctly interpreting research findings.

The current findings suggest that treatment plausibility in research interpretation is neglected by many students and other members of the public. This conclusion is consistent with research showing people neglect base rate information in some situations when estimating probabilities (*e.g.*, Kahneman & Tversky, 1973; Pennycook et al., 2014). For the current study, we deliberately created what to us seemed to be an extremely implausible treatment (Psychic Reinforcement Therapy) to test the limits of this neglect. Apparently, our example could have been even more fantastic, because many people seemed to think that psychic therapy might work. If so, they may accept other implausible ideas said to have “significant” effects.

To improve student understanding and appreciation of treatment plausibility, there are several instructional approaches that instructors might try. One direct tactic is to ask students to explicitly discuss how their interpretation of research findings incorporates theory or prior research. Instructors can also include treatment plausibility on their list of factors to consider when illustrating how to interpret research results. As an exercise, students can be asked to critique studies, or summaries of studies, that vary in terms of treatment plausibility. The two standard results articles from the current study could be used for this activity. The basic procedure would be to have some students read the implausible version, while others read the plausible version. Students could then summarize and discuss the ratings given to the two articles (see Supplemental Information for examples).

We also advise instructors to warn students about the dangers of trying to interpret statistical results in the absence of domain specific knowledge. In such cases, it is prudent to get the opinion of experts. To help make this point, instructors may want to give students multiple opportunities to practice interpreting results using examples such as those provided in the Supplemental Information. What about the common situation when experts do not agree on the meaning of a research result? Here, instructors can emphasize that scientific knowledge is often incomplete, and that it is often impossible to arrive at a firm conclusion until more information becomes available. In general, researchers treat new findings as suggestive and preliminary rather than definitive.

Correcting misunderstandings about statistical significance can also enhance a discussion of treatment plausibility. In the current study, 74% of participants agreed or strongly agreed with the statement “In the context of the news article, I understand what the phrase ’statistically significant’ means.” However, several studies show that many students, researchers, and textbook authors actually misinterpret *p* values and statistical significance (*e.g.*, Cassidy et al., 2019; Falk & Greenbaum, 1995; Haller & Krauss, 2002; Oakes, 1986). Even the definition of a *p* value in the publication manual of the American Psychological Association comes up a bit short^2^. 2The APA manual (American Psychological Association, 2020) defines a *p* value as “the probability of obtaining a value as extreme or more extreme than the one obtained” (p. 88). This definition fails to mention the underlying assumption that the null hypothesis is true, which is necessary to calculate any *p* value.In our experience, students often jump to the conclusion that a statistically significant result means the treatment probably worked (or that the null hypothesis probably is not true). If they mistakenly draw that conclusion, it short-circuits a careful consideration of treatment plausibility. We are sympathetic to Lykken’s (1968) view of the role of statistical tests in research: “Statistical significance is perhaps the least important attribute of a good experiment; it is never a sufficient condition for claiming that a theory has been usefully corroborated, that a meaningful empirical fact has been established, or that an experimental report ought to be published” (p. 151).

A key point for students to understand is that when a study finds a statistically significant result, there is a greater chance it is a false alarm if the treatment is implausible. In other words, if a treatment is probably ineffective, a significant result is likely to be a fluke (see, for example Pashler & Harris, 2012; Wacholder et al., 2004, for further discussion of this idea). Diagnostic medical tests provide a useful analogy for teaching interpretation of statistical tests. If the prior odds that you have a disease are very low, then a positive test, like a statistically significant result, is probably a false alarm (but not always). However, if the prior odds are high, a positive test is likely correct.

A final suggestion for instructors is that students might read and discuss articles such as Lykken (1968) and Lilienfeld (2011). Lykken relates an illuminating anecdote about “Rorschach frog responders” to make the point that a positive outcome in one study does not convince researchers that an implausible theory is now suddenly plausible. Lilenfeld’s article discusses issues related to the evaluation of psychological treatments, and concludes, “To separate the wheat from the chaff in the psychotherapy field, we cannot evaluate treatment process or outcome research in a vacuum. As Reverend Bayes would have reminded us, we must consider such data in conjunction with the plausibility of our theoretical models” (p. 10).

The current study suggests the possibility that neglect of treatment plausibility may be one reason students and others draw incorrect inferences from research findings. A reviewer also pointed out that the reverse may be true: misinterpretation of statistical findings may lead people to downplay or ignore treatment plausibility. Further research is needed to clarify the nature of the association between the degree to which people attend to plausibility and how they interpret research findings. In the meantime, instructors, researchers, and writers may be able to help reduce misinterpretations of research findings by emphasizing both treatment plausibility and correct interpretation of statistical results.

Another important question for future research is whether results like the current findings would occur using actual news or research articles, perhaps on a variety of topics. Also, the teaching suggestions given above should be tested empirically to assess their effectiveness. Further research should also test the extent to which the current findings depend on the wording of the question used to assess the main dependent measure (therapy effectiveness ratings). In the present study, the question was “Based on the information in the article, how likely do you think it is that the therapy helped people lose weight?” A reviewer noted that some participants may have interpreted the phrase “Based on the information in the article” literally, in the sense of “Based *only* on the information in the article, *and no other information*.” For example, consider a participant who was in the psychic therapy-standard results condition. That person may have known there is little evidence for psychic phenomena but chose to ignore that knowledge because it was not explicitly stated in the article. Future research should compare the results of including, and excluding, such a phrase in the questionnaire. For example, an alternate condition could use wording such as “After reading the information in the article.”

According to Sagan (1995), science is characterized by “an essential balance between two seemingly contradictory attitudes—an openness to new ideas, no matter how bizarre or counterintuitive, and the most ruthlessly skeptical scrutiny of all ideas, old and new” (p. 304). New ideas are vital for scientific progress, but plausibility judgments are a key component of the skeptical scrutiny that prevents flawed ideas from gaining undeserved prominence and slowing that progress.

## Supplemental Information

10.7717/peerj.12532/supp-1 Supplemental Information 1 Summary of supplemental analyses

10.7717/peerj.12532/supp-2 Supplemental Information 2 Questionnaire items and class activity material

## Additional Information and Declarations

### Data Availability

The following information was supplied regarding data availability: The data file is available at the Open Science Framework: Thompson, Burt. 2021. “Acceptance of Anomalous Research Findings: Explaining Treatment Implausibility Reduces Belief in Far-Fetched Results.” OSF. June 30. osf.io/c4yhs/
