# Supplementary material for: Acceptance of anomalous research findings: explaining treatment implausibility reduces belief in far-fetched results
Source: PeerJ. 2021 Nov 23;9:e12532. doi: 10.7717/peerj.12532 (PMC8621712; doi:10.7717/peerj.12532)
Supplement: Supplemental Information 1 [file peerj-09-12532-s001.docx]

| **Table S1**  Prior probabilities, posterior probabilities, and inclusion Bayes factors for each effect on therapy effectiveness ratings in the exploratory ANCOVA. | | | |
| --- | --- | --- | --- |
| Effects | P(incl) | P(incl \| data) | BF_incl_ |
| Therapy type | 0.600 | 1.000 | 906344 |
| Results type | 0.600 | 1.000 | 8.600 × 10^6^ |
| Psychic score | 0.500 | 1.000 | 1.784 × 10^12^ |
| Therapy score | 0.500 | 0.992 | 117.664 |
| Statistics experience | 0.500 | 0.124 | 0.141 |
| Therapy type × Results type | 0.200 | 0.862 | 25.062 |

| **Table S2**  ANCOVA for therapy effectiveness ratings with covariates of psychic score, therapy score, and statistics experience. | | | | | | |
| --- | --- | --- | --- | --- | --- | --- |
|  | SS | df | MS | *F* | *p* | η^2^_p_ |
| Therapy type | 15304.57 | 1 | 15304.57 | 30.23 | < .001 | 0.05 |
| Results type | 20090.88 | 2 | 10045.44 | 19.84 | < .001 | 0.06 |
| Therapy type × Results type | 5841.10 | 2 | 2920.55 | 5.77 | 0.003 | 0.02 |
| Psychic score | 33216.45 | 1 | 33216.45 | 65.61 | < .001 | 0.10 |
| Therapy score | 7195.303 | 1 | 7195.303 | 14.212 | < .001 | 0.02 |
| Statistics experience | 115.91 | 1 | 115.91 | 0.23 | 0.632 | 0.00 |
| Residuals | 299217.43 | 591 | 506.29 |  |  |  |

| **Table S3**  ANOVA for therapy effectiveness ratings. | | | | | | |
| --- | --- | --- | --- | --- | --- | --- |
|  | SS | df | MS | *F* | *p* | η^2^_p_ |
| Therapy type | 15200.67 | 1 | 15200.67 | 26.58 | < .001 | 0.04 |
| Results type | 23676.33 | 2 | 11838.17 | 20.70 | < .001 | 0.07 |
| Therapy type × Results type | 4840.33 | 2 | 2420.17 | 4.23 | 0.015 | 0.01 |
| Residuals | 339666 | 594 | 571.83 |  |  |  |

| **Table S4**  Models for psychic score: Prior probabilities, posterior probabilities, and Bayes factors. | | | | |
| --- | --- | --- | --- | --- |
| Models | P(M) | P(M \| data) | BF_M_ | BF_10_ |
| Null model | 0.200 | 0.820 | 18.184 | 1.000 |
| Therapy type | 0.200 | 0.147 | 0.691 | 0.180 |
| Results type | 0.200 | 0.028 | 0.114 | 0.034 |
| Therapy type + Results type | 0.200 | 0.005 | 0.019 | 0.006 |
| Therapy type + Results type + Therapy type × Results type | 0.200 | 3.886 × 10^-4^ | 0.002 | 4.741 × 10^-4^ |

| **Table S5**  Models for therapy score: Prior probabilities, posterior probabilities, and Bayes factors. | | | | |
| --- | --- | --- | --- | --- |
| Models | P(M) | P(M \| data) | BF_M_ | BF_10_ |
| Null model | 0.200 | 0.621 | 6.559 | 1.000 |
| Therapy type | 0.200 | 0.324 | 1.921 | 0.522 |
| Results type | 0.200 | 0.035 | 0.145 | 0.056 |
| Therapy type + Results type | 0.200 | 0.018 | 0.074 | 0.029 |
| Therapy type + Results type + Therapy type × Results type | 0.200 | 0.001 | 0.005 | 0.002 |
