# Supplementary material for: Acceptance of anomalous research findings: explaining treatment implausibility reduces belief in far-fetched results
Source: PeerJ. 2021 Nov 23;9:e12532. doi: 10.7717/peerj.12532 (PMC8621712; doi:10.7717/peerj.12532)
Supplement: Supplemental Information 2 [file peerj-09-12532-s002.docx]

Supplemental material for Acceptance of Anomalous Research Findings: Explaining Treatment Implausibility Reduces Belief in Far-Fetched Results

**Questionnaire Items**

*Note.* For items 1-5 and 7, the phrase in brackets was used for the no-results articles, in which the study was described as still in progress.

1. The study participants dieted [will diet] for two months.

True/False

2. All of the study participants received [will receive] some form of therapy.

True/False

3. Any differences between the groups were [will be] examined statistically.

True/False

4. As part of therapy, dieters kept [will keep] a journal of dreams they had [have] about food.

True/False

5. The researchers compared [will compare] weight lost in the two groups.

True/False

6. A researcher who is not involved in the study is quoted in the article.

True/False

7. Based on the information in the article, how likely do you think it is that the therapy helped [will help] people lose weight?

0% 10% 20% 30% 40% 50% 60% 70% 80% 90% 100%

8. Please briefly explain why you chose that rating.

9. If you wanted to lose weight, how open would you be to trying this type of therapy?

Definitely would not try it 1 2 3 4 5 Definitely would try it

10. Suppose a friend of yours was trying to lose weight. Would you recommend they try this type of therapy?

Definitely not 1 2 3 4 5 Definitely yes

11. How often do you read research articles on scientific or medical topics?

Never, Rarely, Sometimes, Often

12. How often have you tried to lose weight by dieting?

Never, Rarely, Sometimes, Often

13. How many college or university level courses in statistics have you completed? Include any course in which statistics was either the primary focus or was a substantial part of the course.
 0, 1, 2, 3, 4 or more

14. Please indicate your level of knowledge of statistics.
 I know little or nothing about statistics

I have an elementary knowledge of statistics

I am fairly knowledgeable about statistics

I am very knowledgeable about statistics

I have an expert level of knowledge about statistics

15. How often have you performed (either recently, or in the past) any type of statistical analysis of data?
 Never, Rarely, Sometimes, Often

16. How often have you performed (either recently, or in the past) statistical analysis of data that involves summary statistics such as percentages or averages?

Never, Rarely, Sometimes, Often

17. How often have you performed (either recently, or in the past) statistical analysis of data that includes hypothesis testing and interpretation of statistical significance?

Never, Rarely, Sometimes, Often

18. The news article was difficult to understand.

Strongly disagree, Disagree, Slightly disagree, Slightly agree, Agree, Strongly agree

19. In the context of the news article, I understand what the phrase "statistically significant" means.
 Strongly disagree, Disagree, Slightly disagree, Slightly agree, Agree, Strongly agree

20. I believe psychic phenomena are real and should be studied scientifically.

Strongly disagree, Disagree, Slightly disagree, Slightly agree, Agree, Strongly agree

21. There is such a thing as telepathy (communication directly from mind to mind).

Strongly disagree, Disagree, Slightly disagree, Slightly agree, Agree, Strongly agree

22. Therapy (i.e., "talk therapy") can often help people deal with behavioral and psychological problems.

Strongly disagree, Disagree, Slightly disagree, Slightly agree, Agree, Strongly agree

23. If a friend of mine was depressed, I would recommend he or she seek help from a professional therapists such as a clinical psychologist or psychiatrist.

Strongly disagree, Disagree, Slightly disagree, Slightly agree, Agree, Strongly agree

24. Extra Sensory Perception (ESP) is an unusual gift that many persons have and should not be confused with the elaborate trick of entertainers.

Strongly disagree, Disagree, Slightly disagree, Slightly agree, Agree, Strongly agree

25. Therapy can't really help someone who wants to stop smoking or lose weight.

Strongly disagree, Disagree, Slightly disagree, Slightly agree, Agree, Strongly agree

26. There is very little evidence that therapy helps people change for the better.

Strongly disagree, Disagree, Slightly disagree, Slightly agree, Agree, Strongly agree

27. There is a great deal we have yet to understand about the mind, so it is likely that many phenomena such as Extra Sensory Perception (ESP) will one day be proven to exist.

Strongly disagree, Disagree, Slightly disagree, Slightly agree, Agree, Strongly agree

**Class Activity Materials**

A. Fictitious news articles used in the present study (standard result version): Plausible treatment (cognitive behavioral therapy) and implausible treatment (psychic reinforcement therapy).

**Researchers find new therapy may aid weight loss**

After one month of cognitive behavioral therapy, volunteers lost more weight.

According to a new study, weight-loss therapy for as little as one month may help dieters lose extra pounds. The study compared two groups of 40 volunteers, and was published in *Health Psychology Open Science.*

Scientists at Lewiston University conducted the research to test a new version of cognitive behavioral therapy (CBT). CBT aims to help people change both their eating behavior and how they think about food. The therapist helps the dieter set up a sequence of rewards that can be earned by losing weight. In addition, dieters learn to identify and eliminate irrational beliefs about food that can lead to unhealthy eating behaviors.

The placebo control group tried to follow the same diet, but they received a form of therapy that, similar to treatment with a placebo, is not effective. After 30 days, volunteers who received CBT lost more weight and found junk food less desirable than did the control group. The difference between the two groups was statistically significant for both weight loss and food ratings.

In your opinion, how likely is it (0% to 100%) that the therapy helped people lose weight?

Explain why you gave that rating.

**Researchers find new therapy may aid weight loss**

After one month of psychic reinforcement therapy, volunteers lost more weight.

According to a new study, weight-loss therapy for as little as one month may help dieters lose extra pounds. The study compared two groups of 40 volunteers, and was published in *Health Psychology Open Science.*

Scientists at Lewiston University conducted the research to test a new version of psychic reinforcement therapy (PRT). PRT aims to help people change both their eating behavior and how they think about food. Using the psychic ability of extrasensory perception (ESP), the therapist tries to send diet-enhancing messages directly into the mind of the dieter. The messages are designed to reduce irrational beliefs about food that can lead to unhealthy eating behaviors.

The placebo control group tried to follow the same diet, but they received a form of therapy that, similar to treatment with a placebo, is not effective. After 30 days, volunteers who received PRT lost more weight and found junk food less desirable than did the control group. The difference between the two groups was statistically significant for both weight loss and food ratings.

In your opinion, how likely is it (0% to 100%) that the therapy helped people lose weight?

Explain why you gave that rating.

B. Fictitious research summaries: Plausible experimental manipulation (2 minutes vs. 4 minutes study time) and implausible manipulation (80 seconds vs. 90 seconds study time).

**Read the following summary of a research article:**

This study looked at the effect of study time on recall of words. We tested two groups of 40 people. One group was given 4 minutes to study a list of 20 words. Another group was given 2 minutes to study the same list of words. Ten minutes after study, the groups were asked to recall the words. The 4-minute group recalled significantly more words than the 2-minute group, *t*(78) = 2.20, *p* = .02. These data suggest that additional study time can improve recall of studied information.

Please answer the following questions. Assume the study has no major design flaws.

How likely is it (0% to 100%) that the additional study time improved recall?

Explain why you gave that rating.

**Read the following summary of a research article:**

This study looked at the effect of study time on recall of words. We tested two groups of 40 people. One group was given 80 seconds to study a list of 20 words. Another group was given 90 seconds to study the same list of words. Ten minutes after study, the groups were asked to recall the words. The 90-second group recalled significantly more words than the 80-second group, *t*(78) = 2.20, *p* = .02. These data suggest that additional study time can improve recall of studied information.

Please answer the following questions. Assume the study has no major design flaws.

How likely is it (0% to 100%) that the additional study time improved recall?

Explain why you gave that rating.

C. Fictitious research summaries: Implausible treatment with standard results and interpretive results.

**Read the following summary of a research article:**

This study looked at the effect of an herbal medication called Arnica on memory. Arnica was prepared using a homeopathic process. Using water, Arnica is diluted to the point where none of the original medication remains in the water. According to homeopathic theory, the medication is effective because the subatomic structure of the water changes during the dilution process. In a double-blind, placebo-controlled study, we tested two groups of 50 people. The treatment group received an Arnica pill and the control group received an identical looking placebo pill that contained no active ingredient. Each group then studied a list of 20 words for 5 minutes. After a 10 minute break, the groups were asked to recall the words. On average, participants in the Arnica group recalled more words than the placebo group, and the difference was statistically significant, *t*(98) = 2.79, *p* = .006. These data suggest that homeopathic Arnica can improve recall of studied information.

Please answer the following questions. Assume the study has no major design flaws.

How likely is it (0% to 100%) that the Arnica pill improved recall?

Explain why you gave that rating.

**Read the following summary of a research article:**

This study looked at the effect of an herbal medication called Arnica on memory. Arnica was prepared using a homeopathic process. Using water, Arnica is diluted to the point where none of the original medication remains in the water. According to homeopathic theory, the medication is effective because the subatomic structure of the water changes during the dilution process. In a double-blind, placebo-controlled study, we tested two groups of 50 people. The treatment group received an Arnica pill and the control group received an identical looking placebo pill that contained no active ingredient. Each group then studied a list of 20 words for 5 minutes. After a 10 minute break, the groups were asked to recall the words. On average, participants in the Arnica group recalled more words than the placebo group, and the difference was statistically significant, *t*(98) = 2.79, *p* = .006. Homeopathic theories conflict with well-established scientific medical knowledge, and violate basic laws of physics and chemistry. Statistically significant differences do occur purely by chance. Hence, the results of this study are almost certainly a fluke, and do not support the hypothesis that homeopathic preparations of Arnica improve recall.

Please answer the following questions. Assume the study has no major design flaws.

How likely is it (0% to 100%) that the Arnica pill improved recall?

Explain why you gave that rating.
